# Evaluating the impact of delayed-phase imaging in Contrast-Enhanced Mammography on breast cancer staging: A comparative study of abbreviated *versus* complete protocol

**DOI:** 10.1007/s11547-024-01838-3

**Published:** 2024-07-10

**Authors:** Naomi Calabrò, Flavia Abruzzese, Eleonora Valentini, Anna Clelia Lucia Gambaro, Silvia Attanasio, Barbara Cannillo, Marco Brambilla, Alessandro Carriero

**Affiliations:** 1 SCDU Radiodiagnostica, Ospedale Maggiore Della Carità, 28100 Novara, Italy; 2 Dipartimento Di Medicina Translazionale, Università del Piemonte Orientale, 28100 Novara, Italy; 3 SCDO Fisica Sanitaria, Ospedale Maggiore Della Carità, 28100 Novara, Italy

**Keywords:** Breast cancer, Contrast-Enhanced mammography, Inter-Observer agreement, Breast lesion, Dual-Energy contrast**-**Enhanced spectral mammography

## Abstract

**Purpose:**

Contrast-enhanced mammography (CEM) is an innovative imaging tool for breast cancer detection, involving intravenous injection of a contrast medium and the assessment of lesion enhancement in two phases: early and delayed. The aim of the study was to analyze the topographic concordance of lesions detected in the early- versus delayed phase acquisitions.

**Materials and methods:**

Approved by the Ethics Committee (No. 118/20), this prospective study included 100 women with histopathological confirmed breast neoplasia (B6) at the Radiodiagnostics Department of the Maggiore della Carità Hospital of Novara, Italy from May 1, 2021, to October 17, 2022. Participants underwent CEM examinations using a complete protocol, encompassing both early- and delayed image acquisitions. Three experienced radiologists blindly analyzed the CEM images for contrast enhancement to determine the topographic concordance of the identified lesions. Two readers assessed the complete study (protocol A), while one reader assessed the protocol without the delayed phase (protocol B). The average glandular dose (AGD) of the entire procedure was also evaluated.

**Results:**

The analysis demonstrated high concordance among the three readers in the topographical identification of lesions within individual quadrants of both breasts, with a Cohen’s κ > 0.75, except for the lower inner quadrant of the right breast and the retro-areolar region of the left breast. The mean whole AGD was 29.2 mGy. The mean AGD due to CEM amounted to 73% of the whole AGD (21.2 mGy). The AGD attributable to the delayed phase of CEM contributed to 36% of the whole AGD (10.5 mGy).

**Conclusions:**

As we found no significant discrepancy between the readings of the two protocols, we conclude that delayed-phase image acquisition in CEM does not provide essential diagnostic benefits for effective disease management. Instead, it contributes to unnecessary radiation exposure.

## Introduction

Breast cancer is the most common neoplasm among women, accounting for 41%, 36%, and 21% of all cancer diagnoses within the age cohorts of 0–49, 50–69, and > 70 years, respectively [1, 2]. It remains the primary cause of cancer-related mortality among women globally [3]. In Italy, breast cancer is responsible for 28% of oncological fatalities in women under 50, decreasing to 21% between the ages of 50 and 69, and 14% beyond 70 years [4]. However, mortality rates have recently been declining across all age groups [4], especially in women under 50, due to the growing implementation of early diagnosis programs and therapeutic advancements. Currently, the 5-year survival rate following diagnosis stands at 87%, extending to 80% over 10 years [4].

Early-stage breast carcinoma is typically asymptomatic and is usually detected through preventative screening measures. Biennial mammography is the recommended screening protocol for women aged 50 to 69 years [5]. A major challenge in breast imaging today is the enhancement of diagnostic sensitivity and specificity, particularly in women with dense breast tissue [6–8], comprising 43% of women between 40 and 74 years of age. In these patients, the sensitivity of conventional mammography decreases from 85to 47.8% [5, 9].

**Fig. 1:**
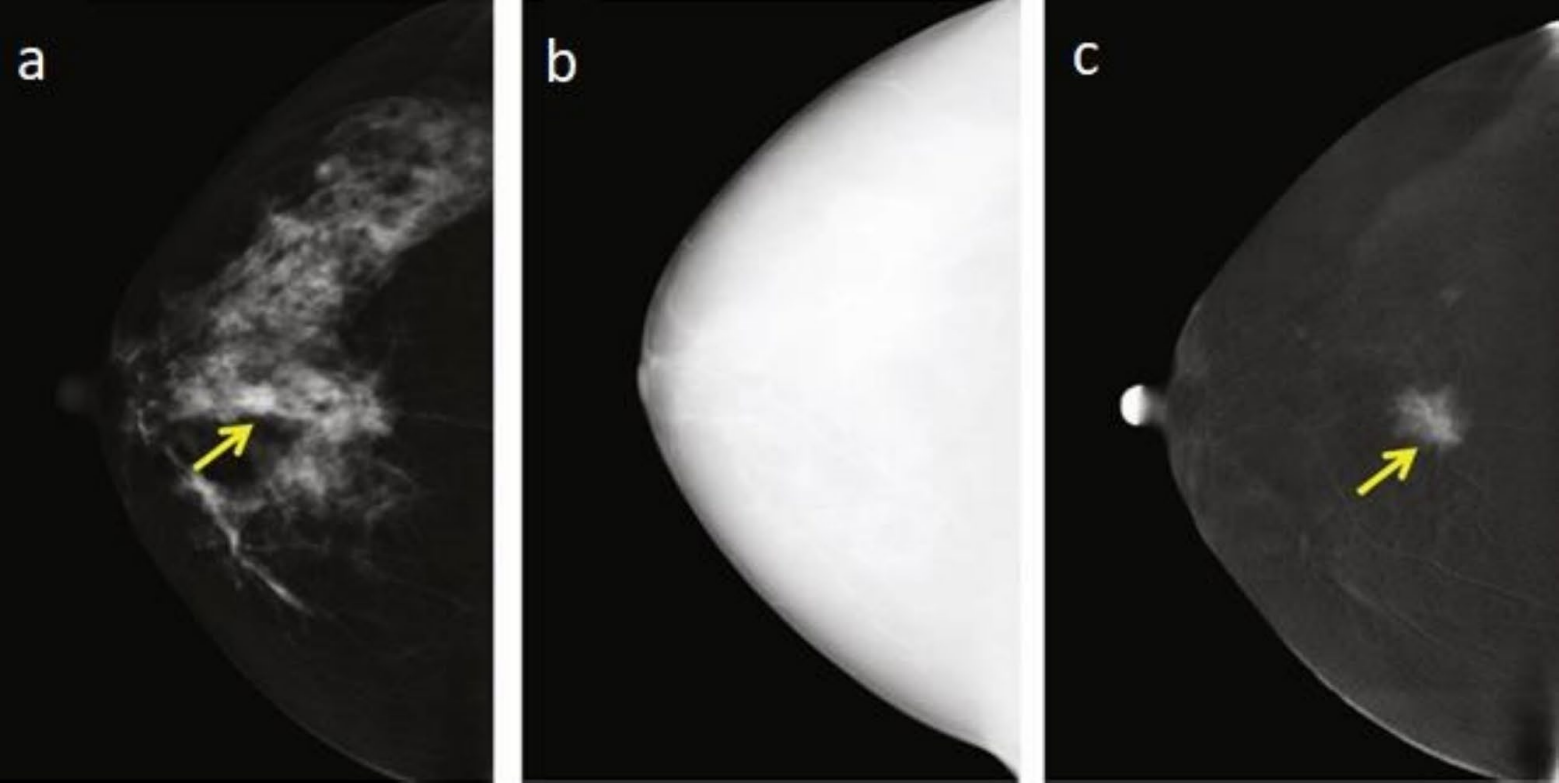
Technical representation of dual-energy contrast-enhanced spectral mammography (CEM). **a** Low- energy acquisition equivalent to conventional mammography. **b** Non-diagnostic high-energy acquisition. **c** Spectral subtraction. The yellow arrows in (a) and (c) indicate a breast lesion

Within the context of technological advancements in mammography, contrast-enhanced mammography (CEM) has recently been optimized [10] and is emerging as an alternative method to magnetic resonance imaging (MRI) [11–13]. More specifically, dual-energy contrast-enhanced spectral mammography (CEM) is a diagnostic technique involving the injection of contrast medium followed by a dual-phase imaging at 2 min (early phase) and 8 min (delayed phase) post-injection, with subsequent subtraction of the acquired data (Fig. 1) [14–16].

The primary indications for CEM are the pre-operative staging to ascertain multifocality, multicentricity, and/or bilaterality, as well as evaluating the response to neoadjuvant chemotherapy (NAC) in patients with breast neoplasms [13, 14]. In the preoperative staging using CEM, some studies [13] have shown a slight discrepancy between the tumor size measured by CEM and the actual histopathological tumor size, ranging from 0.03 to 5 mm. This variance is typically addressed during surgical procedures by extending the incision to ensure disease-free margins.

Based on the above information, the aim of this study was to assess whether delayed- *versus* early-phase acquisition provides additional information in identifying areas of pathological enhancement in patients with a known breast pathology.

## Materials and methods Patients

Following approval from the Ethics Committee No. 118/20, this study was conducted at the Radiodiagnostics Department of the Maggiore della Carità Hospital in Novara and the University of Eastern Piedmont, Italy, from May 1, 2021, to October 17, 2022. A total of 100 women, with an average age of 57.5 years (range 33–82 years) ± 12 ds, all of whom had histologically confirmed breast cancer and were scheduled for surgical intervention, were consecutively and prospectively enrolled.

Inclusion criteria were as follows:

- Women scheduled for surgery with a histologically confirmed invasive breast carcinoma (T1–2);
- Age > 30 years;
- Provision of written informed consent;
- Negative medical history for adverse events related to the use of iodinated contrast agents;
- Normal renal function, verified by creatinine and glomerular filtration rate measurements.

Exclusion criteria were as follows:

- Women with breast implants;
- Age ≤ 30 years;
- Positive medical history for severe adverse events related to the use of iodinated contrast agents;
- Impaired renal function.

### CEM technique

CEM was performed using the Selenia Dimensions Mammography System® (Hologic, USA). Before conducting CEM, venous access was established in the forearm, through which a low-osmolarity iodinated contrast agent (Iomeron 350) was administered in a single dose using an automatic injector (Bracco Injeneering Empower CTA^+^), at an injection rate of 2–3 ml/sec, followed by a saline flush of 20 ml. Intravenous contrast agent injection was performed with the patient in a seated position.

The administered contrast volume was calculated at 1.5 ml/kg of body weight, up to a maximum of 110 ml. Two min post-injection, dual-energy mammographic projections in cranio-caudal (CC) and medio-lateral oblique (MLO) views were acquired for each breast. The examination was completed with delayed acquisitions in CC and MLO projections for each breast, starting from the 7th min following contrast agent administration.

### Radiological image analysis

To assess the contribution of the delayed-phase acquisition in identifying suspicious lesions, three experienced breast radiologists (R1, R2, R3) in service near the Breast Unit section for a minimum of ten years, and for a minimum of two years in the CEM section (since May, 1, 2021) conducted a blind review the CEM images. Although aware of the existence of a histologically confirmed primary tumor, these radiologists were charged with identifying areas of contrast enhancement that suggested a unifocal lesion or raised suspicion of multifocality, multicentricity, and/or bilaterality.

For precise topographical localization of lesions, the breast gland was segmented into five quadrants: upper outer quadrant (UOQ), lower inner quadrant (LIQ), upper inner quadrant (UIQ), lower outer quadrant (LOQ), and retro-areolar area.

In assessing CEM enhancement, the readers employed the ACR BI-RADS® 2022 scale (1 = negative; 2 = benign; 3 = probably benign; 4 = probably malignant; 5 = highly suspicious). For analysis purposes, this scale was simplified into a binary measure, where 0 denoted no lesion or benign lesion (BI-RADS 1/2) and 1 indicated a suspicious or highly suspicious lesion (BI-RADS 3/4/5). This dichotomous approach was adopted because the radiologists, already aware of the presence of the malignant lesion, seldom assigned intermediate values (BI-RADS 3 and 4).

**Fig. 2:**
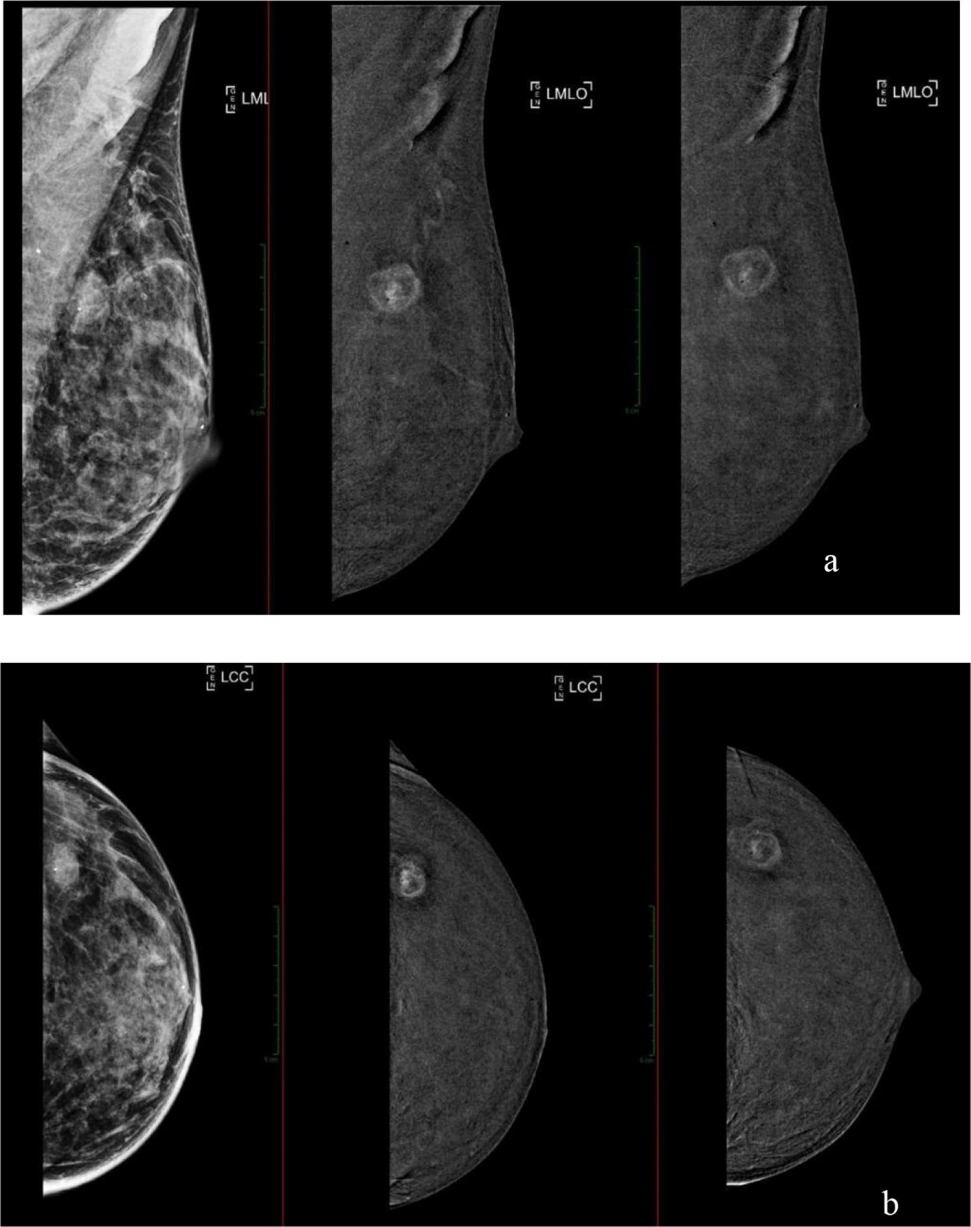
Protocol A: Low-energy (LE) image and recombined image in the early phase, as well as subtracted image from the delayed-phase acquisition in **a** left mediolateral oblique (LMLO) or **b** left craniocaudal (LCC) view

In the evaluation process, two breast radiologists blindly reviewed the entire set of examinations, including low-energy (LE) images and the recombined images (RI) acquired in both the early and delayed phases (protocol A) (Fig. 2).

**Fig. 3:**
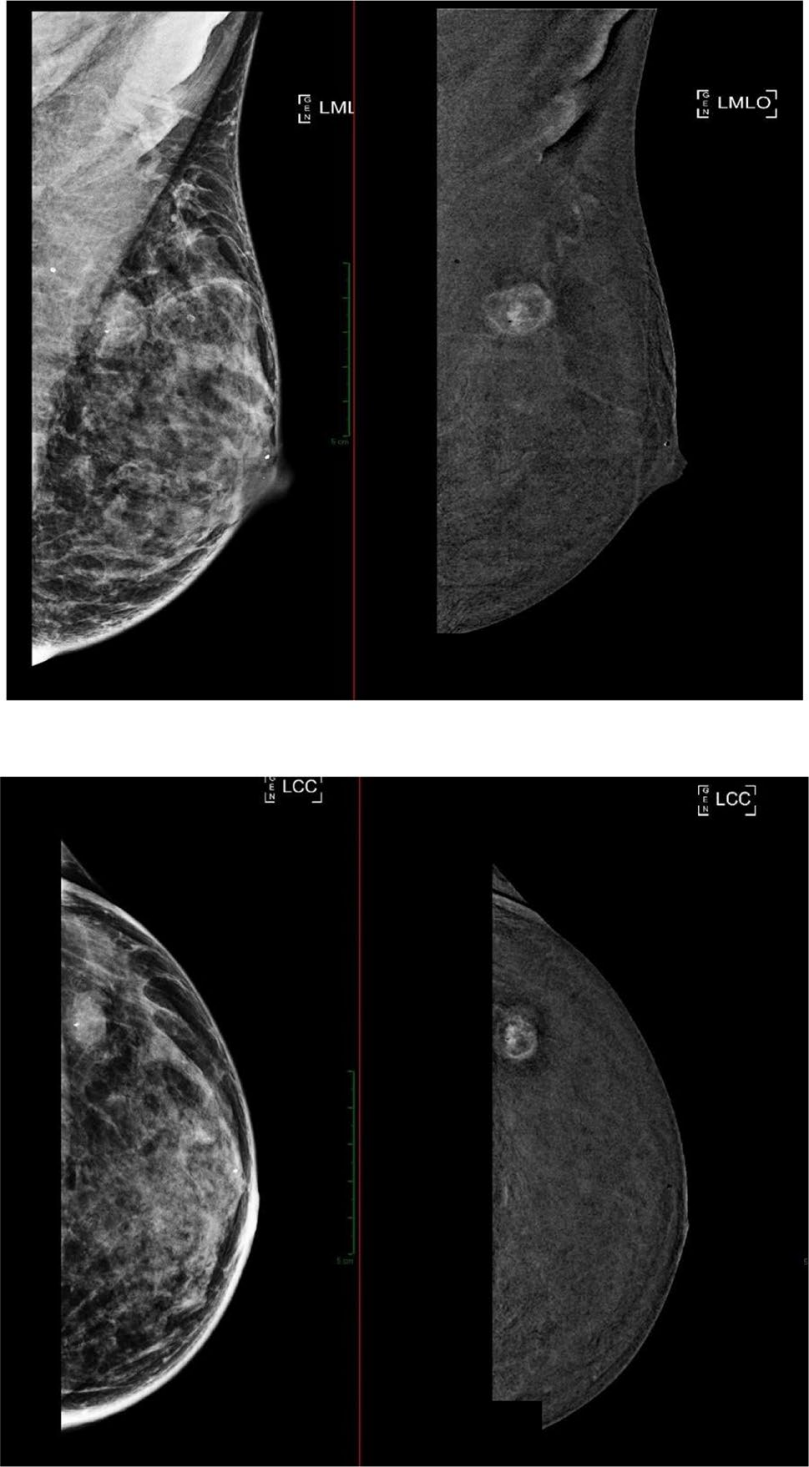
Protocol B: early-phase acquisition followed by subsequent subtracted image in **a** left mediolateral oblique (LMLO) or **b** left craniocaudal (LCC) view

The third breast radiologist blind-reviewed only the LE images and contrast-enhanced acquisitions from the early phase, excluding the delayed-phase acquisitions (protocol B) (Fig. 3).

The assessments provided by the three readers were then statistically compared to evaluate the reproducibility between the first two radiologists, who assessed the full examination under protocol A, and between these radiologists and the third, who only reviewed early-phase acquisitions under protocol B.

### Dosimetry

The Medical Physics Department analyzed 60 examinations comprising 4 tomosyntheses (TOMO) (2 for each breast in CC and MLO projections) and 4 dual-energy mammograms (MLO and CC) for each breast, acquired after contrast medium injection in both “early” and “delayed” modes, resulting in 16 2D acquisitions and four 3D acquisitions per patient. The overall mean glandular dose (AGD) of the protocol, as well as individual AGD values related TOMO and CEM projections, was evaluated.

The dosimetric information (AGD) was retrieved by the local Dose Monitoring System (Graydetector, Elco). The accuracy of the displayed AGD is routinely checked in the quality control program and maintains an accuracy within 10%.

### Statistical analysis

The reproducibility of lesion scoring between two readers was evaluated using the Cohen's κ-coefficient, which is expressed as a number between 0 and 1, where 0 indicates no reproducibility, and 1 represents perfect agreement, with values > 0.75 generally indicative of good reproducibility. To ease the interpretation of κ in terms of disagreement between the two observers, a 95% confidence interval (CI) was adopted.

The distribution of κ values was assumed to be approximately normal. To test the significance of the differences between two independents κ values, a normal curve test was applied using the following formula:

$$\text{Z }=\frac{{K}_{1}- {K}_{2}}{\sqrt{{\sigma }_{K1}^{2}-{\sigma }_{K2}^{2}}}$$

A *p*-value < 0.05 was considered statistically significant.

## Results

The study analyzed 100 patients with histologically confirmed breast cancer (Table 1).

**Table 1:** Histopathology of the carcinomas included in the study

Carcinoma histology (n = 100)	n
Invasive ductal carcinoma	75
Invasive lobular carcinoma	19
Serous carcinoma	2
Mucinous carcinoma	4

All patients consented to undergo CEM to assess the potential presence of multicentricity, multifocality, and/or bilaterality. CEM successfully identified 58 cases of unifocality, 11 of multifocality, 26 of multicentricity, and 5 of bilaterality (Table 2). Of note, the total count of lesions identified was 101 lesions in 100 patients, due to one patient presenting as bilateral prior to CEM application (Table 2).

**Table 2:** Classification of radiological pattern at pre-operative staging after CEM in patients with histologically confirmed lesions

Pattern	Patients n (%)
Unifocal	58 (58)
Multicentric	11 (11)
Multifocal	26 (26)
Bilateral	6 (6)

**Fig. 4:**
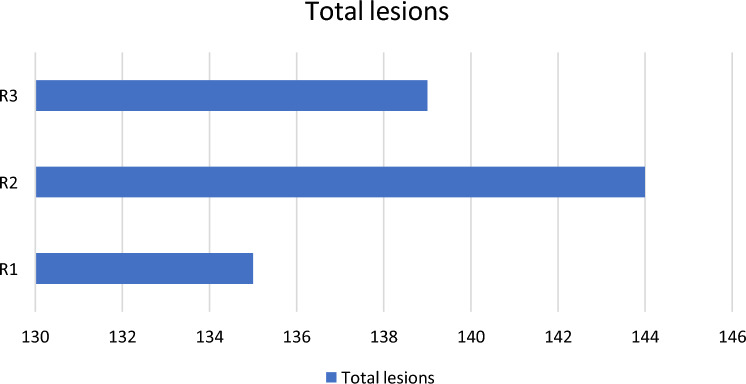
Number of total lesions identified by individual radiologists

In terms of lesion identification, the readers utilizing the full CEM protocol (Radiologists 1 and 2; R1, R2) identified 135 and 144 areas of contrast enhancement indicative of breast cancer, respectively, which included the 101 biopsy-confirmed lesions. Conversely, the third reader (Radiologist 3, R3), who followed the protocol excluding the delayed phase (B), detected 139 areas of contrast enhancement suspected of breast cancer, also encompassing the 101 biopsy confirmed lesions (Fig. 4).

**Fig. 5:**
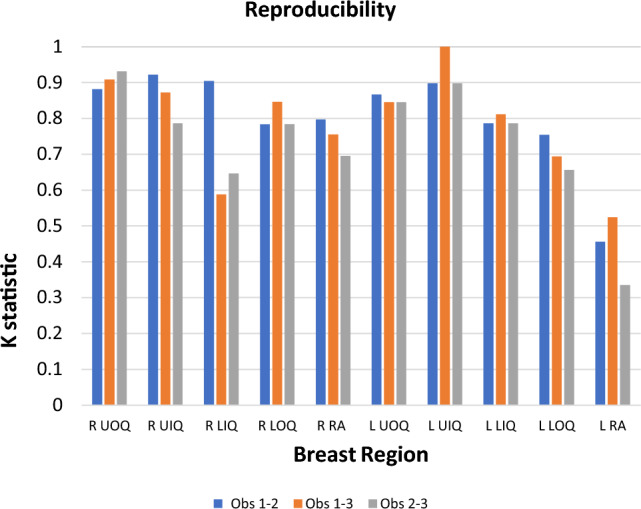
Bar chart depicting the reproducibility analysis among the three observers reported in Table 3

The agreement on the topographical localization of lesions within individual quadrants of both breasts was notably high among the three readers showing a high level of concordance in each quadrant, with κ value > 0.75 in each quadrant, except for the right LIQ and the retro-areolar region on the left side. In these particular quadrants, discrepancies were observed. In particular, the agreement between R1 and 2, who had analyzed the complete examination, was higher compared to the agreement between them and R3, who did not review the delayed phase images. The κ values were 0.59 (CI: 0.24–0.94) and 0.65 (CI: 0.33–0.97), respectively. The level of agreement in the retro-areolar region on the left was low across all three readers. However, these discrepancies were not statistically significant and concerned only the location, not the malignant nature of the lesions, about which there was unanimous agreement among the readers (Fig. 5, Table 3).

**Table 3:** Reproducibility analysis by Cohen's κ (concordance index) with 95% CI (confidence interval) between R1 and 2, R1 and 3, and R2 and 3

QUADRANT	κ 1,2(95%CI)	κ 1,3(95% CI)	κ 2,3(95%CI)
R UOQ	0.88 (0.78–0.98)	0.91 (0.82–1.00)	0.93 (0.85–1.00)
R UIQ	0.92 (0.81–1)	0.87 (0.73–1)	0.79 (0.60–0.97)
R LIQ	0.90 (0.72–1)	0.59 (0.24–0.94)	0.65 (0.33–0.97)
R LOQ	0.78 (0.54–1)	0.85 (0.64–1)	0.78 (0.54–1)
R RETROAREOLAR	0.78 (0.60–0.99)	0.76 (0.52–0.99)	0.70 (0.46–0.93)
L UOQ	0.87 (0.73–0.97)	0.85 (0.73–0.96)	0.85 (0.73–0.96)
L UIQ	0.99 (0.76–1)	1 (1)	0.90 (0.76–1)
L LIQ	0.79 (0.60–0.97)	0.81 (0.63–0.99)	0.79 (0.60–0.97)
L LOQ	0.75 (0.54–0.96)	0.69 (0.46–0.92)	0.66 (0.41–0.90)
L RETROAREOLAR	0.46 (0.09–0.81)	0.52 (0.12–0.93)	0.34 (0–0.81)

UOQ = Upper outer quadrant; LIQ = Lower inner quadrant; UIQ = Upper inner quadrant;

LOQ = Lower outer quadrant; R = right; L = left

Complete concordance was observed among all three readers regarding the presence of multifocality, with a κ value of 1. This analysis revealed that 26 patients exhibited multiple lesions within the same breast.

**Fig. 6:**
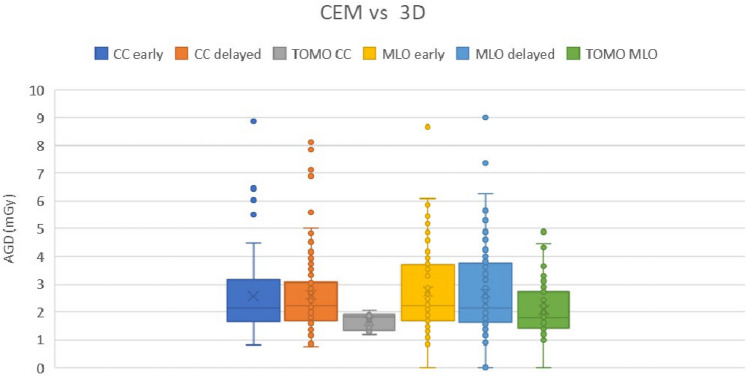
Box-and-whisker plot showing the comparison, for each projection, between contrast-enhanced subtraction mammography (CEM) and tomosynthesis (TOMO)

Table 4 illustrates the AGD contributions by projection and modality, while Fig. 6 presents the AGD per patient attributed to CEM for both early and delayed phases, and to tomosynthesis. This data highlights the dosimetric impact of each modality and phase in clinical settings.

**Table 4:** Average glandular dose (AGD) among different projections (CC and MLO) and AGD from 16 acquisitions

	Average (mGY)	Median (mGY)	St. dev (mGY)	Min (mGY)	Max (mGY)
CC	10.2	9.1	4.8	3.6	26.2
MLO	10.9	9.3	5.0	3.8	21.4
CC + MLO	21.1	17.6	9.5	7.9	46.7

The mean AGD for each 2D modality, whether early or delayed, was similar (2.20 mGy and 2.22 mGy, respectively). These values were notably higher than those observed for three-dimensional (3D) tomosynthesis acquisitions, which averaged at 1.7 mGy.

## Discussion

CEM stands as a cutting-edge technique in breast imaging that has significantly expanded the diagnostic possibilities for breast cancer. Breast cancer diagnosis. Numerous studies [16, 17] indicates that CEM can fulfill similar diagnostic roles to breast MRI, particularly due to its ability to assess suspicious breast lesions by capitalizing on tumor neoangiogenesis through kinetic enhancement. This capability allows CEM and contrast-enhanced breast MRI to surpass traditional mammography in effectiveness, especially in patients with dense breast tissue [14].

To date, extensive blinded inter-observer studies have demonstrated the potential of CEM to enhance breast carcinoma diagnosis, exhibiting superior sensitivity, specificity, positive predictive value, negative predictive value, and overall accuracy [14]. However, only a few studies have specifically assessed the contribution of delayed-phase acquisition in CEM. Among these [17, 18], some have sought to distinguish malignant masses from benign ones through quantitative analysis of contrast enhancement curves, mirroring approaches used in MRI models. Results indicate that malignant lesions typically exhibit more pronounced contrast uptake than benign ones, showing a moderate level of concordance with MRI enhancement patterns. These insights suggest that quantitative analysis of enhancement kinetics characteristics could become a standard practice in CEM clinical evaluations.

In this context, it is imperative to acknowledge that the dual-energy CEM technique requires two exposures for each projection, which inherently impacts radiation dosage. Recent studies have revealed that the radiation dose with early-phase acquisition alone increases by more than 60–70% compared to standard 2D mammography [13] This increase is influenced by various factors, including the type of imaging equipment, system settings, and breast thickness [13, 14].

At our institution, the Medical Physics Department analyzed 60 CEM protocols, involving the acquisition of 4 tomosynthesis (TOMO) images (2 for each breast in CC and MLO projections) and 4 dual-energy mammograms per breast (MLO and CC) following contrast medium infusion in both “early” and “delayed” modes, resulting in 16 2D acquisitions per patient, cumulatively amounting to 952 acquisitions. This analysis also consisted in evaluating the overall AGD of the protocol and the individual AGD values related to TOMO and CEM projections (Fig. 6).

The comprehensive protocol at our institution, which includes TOMO in both projections (CC and MLO) and the two CEM acquisitions (*i.e.*, early and delayed), resulted in an AGD of approximately 29.2 mGy, equivalent to an effective dose of about 3.5 mSv. Notably, the delayed acquisitions accounted for 36% of this dose. However, these dose levels remain below within the acceptable limits set by international regulations [15] and the Mammography Quality Standards Act guidelines [16], which state that the dose increase does not constitute a significant lifetime risk factor.

In alignment with the core principles of radiation protection—justification, optimization, and dose limitation—the objective of our study was to evaluate the contribution of delayed-phase CEM acquisition in preoperative locoregional and bilateral staging of breast cancer, while aiming to minimize dose exposure. To this end, we examined the inherent variability between two experienced breast radiologists who interpreted a complete protocol (A) and juxtaposed it with the variation observed between these two radiologists and the radiologist who had access to the protocol without the delayed phase (B). Our statistical analysis, using the Cohen’s κ-coefficient statistic, revealed no significant differences in diagnostic outcomes. Similarly, the reproducibility index showed consistency across all breast quadrants, with no statistically significant distinctions.

**Fig. 7:**
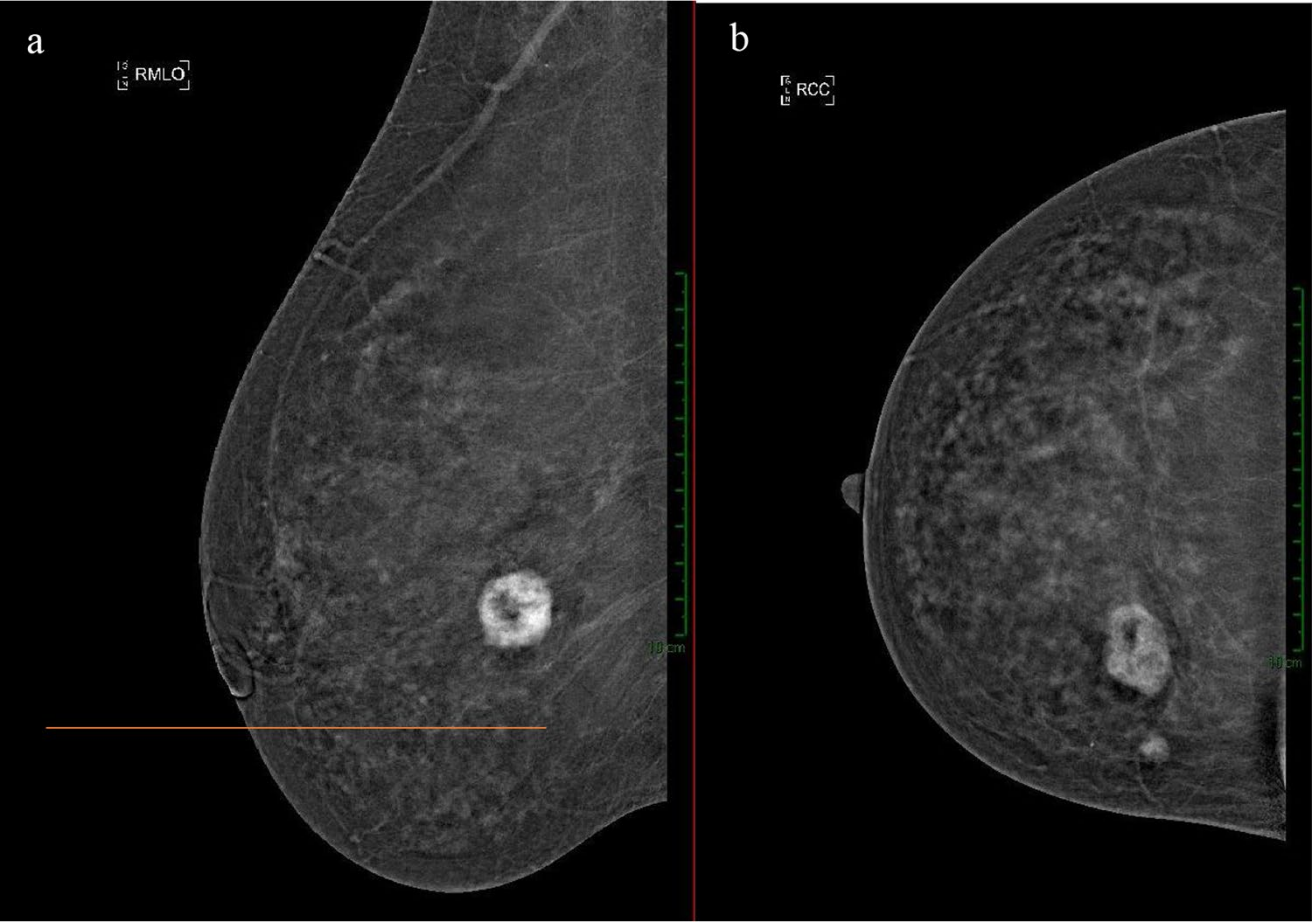
Example of a right-sided lesion in the MLO projection **a** between the upper and lower quadrants, while in the CC projection **b**, it appears within the inner quadrants

**Fig. 8:**
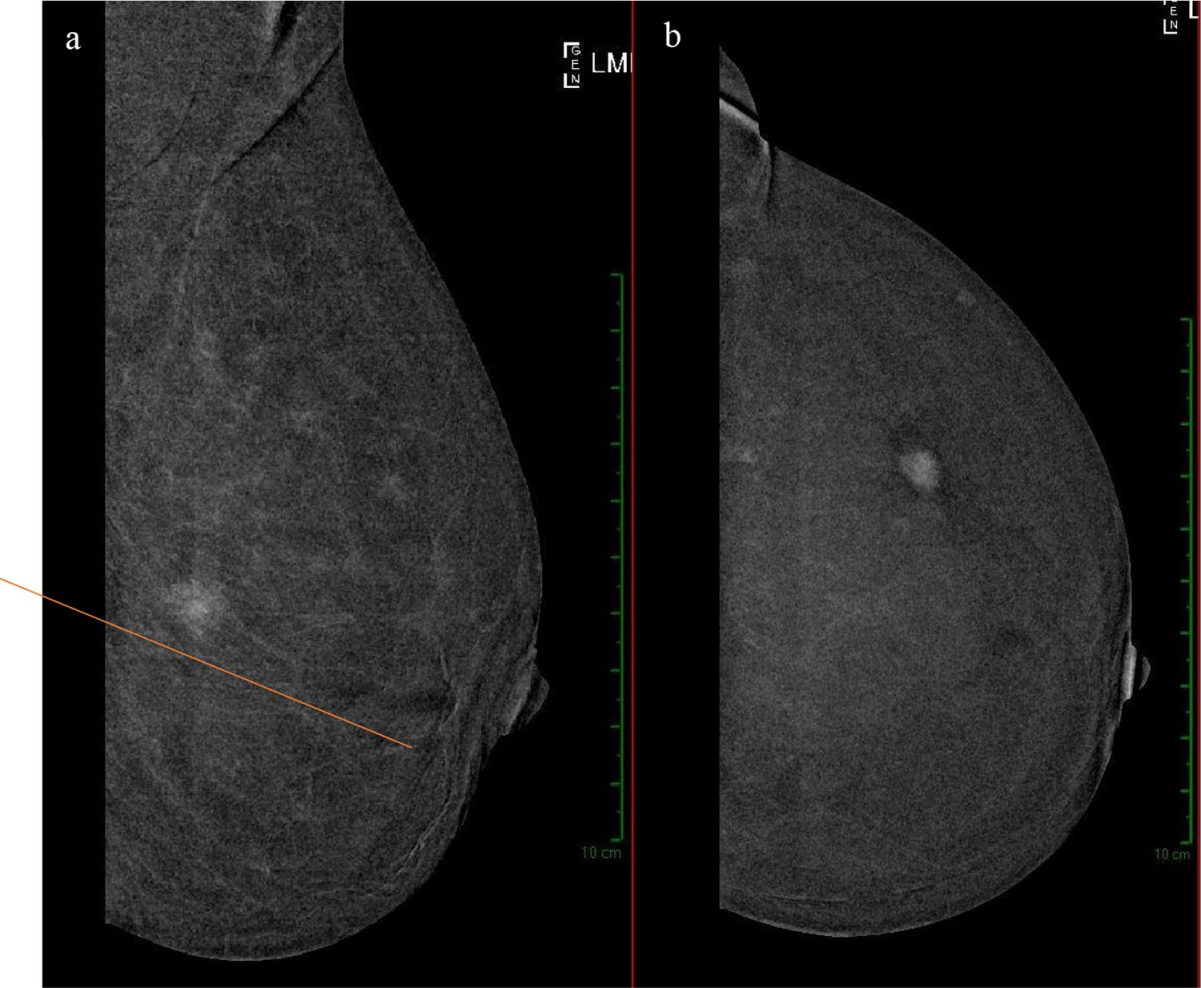
Example of a left-sided lesion in the MLO projection **a** between the upper and lower quadrants, while in the CC projection **b** appears in the outer quadrants

As shown in Fig. 7, there is a noticeable but statistically insignificant divergence among observers in two cases, likely due to localization challenges. Specifically, in the right LIQ, discordance arises between the first two readers and the third one. This discordance was mainly due to difficulties in accurately pinpointing lesions positioned between two quadrants, an issue deriving from the initial quadrant division which lacked provision for “quadrant crossover.” In the MLO projection, where the lesions projected along the nipple line region (Fig. 7, 8), only an ultrasound examination could have provided a more precise localization of the lesions. The observed discrepancy might also be influenced by the fact that the delayed phase involves additional repositioning and compression of the breast, which is not necessarily replicated in the early phase, aiding the reader in better localizing the lesion.

A similar scenario was noted in the retro-areolar region on the left side, where discrepancies were observed for all three readers. Nevertheless, it is important to point out that these discrepancies were not statistically significant and often involved interindividual interpretation, which does not align with a precise quadrant division.

Despite the valuable insights provided, our study has several limitations. Firstly, CEM was not routinely performed for all breast cancer cases as it is not a mandatory modality for clinical management. However, the cases analyzed in our study were selected consecutively from patients with histologically confirmed lesions. Secondly, the study involved a limited number of patients, which may impact the generalizability of the findings. Thirdly, the initial strategy of dividing the breasts into five quadrants made it difficult to localize lesions that were positioned between two quadrants in mammography, complicating accurate mammographic localization.

Despite these limitations, our study indicates that delayed-phase acquisition of CEM does not provide critical additional value for locoregional staging and management of breast cancer patients.

## Conclusions

Our study demonstrates that addition of late-stage acquisition in CEM does not enhance reader concordance compared to early-stage acquisition for loco-regional staging of breast cancer. This finding suggests that the additional imaging phase may not provide significant diagnostic benefits in this specific context.

We acknowledge that the study has some limitations, primarily related to the fact that CEM is not currently used as a routine examination for all patients with breast lesions but is selectively used for those with histologically confirmed breast lesions. This selective application contributed to the relatively small sample size of our study, potentially limiting the generalizability of our findings. In addition, the division of the breast into five quadrants, including the retro-areolar region, presented challenges in lesion localization, leading to increased discordance among readers. Despite these limitations, the results of our study are encouraging and provide a valuable foundation for future research.

Our investigation represents one of the first to systematically evaluate the impact of late-phase acquisition within the CEM protocol. Moving forward, studies with larger sample sizes and more diverse patient populations are essential to further validate our findings and refine the clinical application of CEM, optimizing its utility in the diagnosis and management of breast cancer.
